# Age Estimation Based on Children's Voice: A Fuzzy-Based Decision Fusion Strategy

**DOI:** 10.1155/2014/534064

**Published:** 2014-06-05

**Authors:** Seyed Mostafa Mirhassani, Alireza Zourmand, Hua-Nong Ting

**Affiliations:** Biomedical Engineering Department, Faculty of Engineering, University of Malaya, Lembah Pantai, 50603 Kuala Lumpur, Malaysia

## Abstract

Automatic estimation of a speaker's age is a challenging research topic in the area of speech analysis. In this paper, a novel approach to estimate a speaker's age is presented. The method features a “divide and conquer” strategy wherein the speech data are divided into six groups based on the vowel classes. There are two reasons behind this strategy. First, reduction in the complicated distribution of the processing data improves the classifier's learning performance. Second, different vowel classes contain complementary information for age estimation. Mel-frequency cepstral coefficients are computed for each group and single layer feed-forward neural networks based on self-adaptive extreme learning machine are applied to the features to make a primary decision. Subsequently, fuzzy data fusion is employed to provide an overall decision by aggregating the classifier's outputs. The results are then compared with a number of state-of-the-art age estimation methods. Experiments conducted based on six age groups including children aged between 7 and 12 years revealed that fuzzy fusion of the classifier's outputs resulted in considerable improvement of up to 53.33% in age estimation accuracy. Moreover, the fuzzy fusion of decisions aggregated the complementary information of a speaker's age from various speech sources.

## 1. Introduction

Speaker age has attracted considerable attention among researchers studying recent applications of speech processing. Speaker age provides valuable information that can also improve the performance of automatic speech recognition (ASR) systems as well [1, 2]. Many systems that employ speech data demand a type of user adaptation system that can be adapted with the age of a user. Additionally, in speech synthesis, the appropriate language model can be properly selected based on the age information of the speaker. In commercial applications such as advertising, the target age group can be effectively selected based on speaker's age estimation. Moreover, in ASR systems, the underlying model can be adaptively selected to improve the speech recognition rate.

The estimation of a speaker's age is often performed based on groups of speakers in groups with a wider age range; however, few studies have conducted estimations based on children's speech. In this paper, the problem of age estimation in the context of children speech is addressed. In the diagnosis of some speech disorders, including dyslexia, the estimation of children's age provides valuable information [3, 4]. Moreover, in some interactive educational computer games [5–8], speech-based age estimation plays an important role in adapting systems to their users.

Based on different acoustical features and classifiers, a large number of methods for evaluation of speaker's age have been proposed in literature [2, 9, 10]. Common features of such systems include using hidden Markov models (HMM) [11], support vector machines [12–14], and Gaussian mixture model (GMM) [2] and improvement of the age classes based on data projection to lower spaces [1, 15]. Iseli et al. [1] modeled speakers by HMM weight supervector. Afterwards, to decrease the dimension of the input space, they employed a weighted supervised nonnegative matrix factorization. Age of speakers has also been estimated based on least squares support vector regression. Harnsberger et al. [16] investigated fundamental frequency and speaking rate to distinguish younger male speakers from older male speakers. Dobry et al. [15] reduced feature dimensions by weighted-pair wise principal components analysis based on the nuisance attribute projection. Using SVM to classify the features, they reported up to 10% improvement of the accuracy via the proposed dimension reduction. Mahmoodi et al. [12] used an SVM with RBF kernel, which received Mel-frequency cepstral coefficients (MFCC) and PLP coefficients as features. They repeated the experiments for different numbers of MFCCs. Bahari and his colleagues modeled the speakers' utterances by their corresponding *i*-vectors then they employed a support vector regressor to estimate the age of the speakers [17]. Müller and Burkhardt [9] proposed an age and gender estimation method based on a combination of regression and classification. They performed combination using the posterior probability of an SVM based regressor trained depending on the speaker's age and a gender classifier. Van Heerden et al. [13] employed a GMM to provide a supervector for SVM. Afterwards, they used the SVM with three different kernels in order to estimate the age and gender of the speakers. Li and his colleagues [18] proposed a method for identification of gender and age of the speakers based on acoustic and prosodic level information fusion. They employed large number of subsystems including SVM based on 450-dimensional utterance level features including acoustic, prosodic, and voice quality information, MFCC features, and sparse representation based on UBM weight posterior probability supervectors.

In statistical modeling of the age estimation systems, each hypothesis (classifier) has its own advantages. At the same time, performance of the classifiers in modeling such systems depends not only on the classification methods but also on the processing data. Modeling of complicated distribution of training data in *n*-dimensional feature space requires the use of higher order of nonlinearity or more complex modeling method. Such complexity results in problems that include overfitting of the classifiers. To cope with this problem, some approaches divided the complex problem into some simpler ones [19]. For this purpose, the processing data can be separated into subgroups so that a less complicated modeling method can efficiently handle the classification of each subgroup data. Through this approach, the fusion of decisions made by each preliminary classifier can be used to determine the overall classification results.

Fusion of information has been proposed in literature. For example, Benediktsson [20] introduced a multisource classifier based on a combination of a number of statistical classifiers. In this method, two preliminary classifiers trained with different sources are used to assess the membership of testing samples. In case of agreement of the classifiers on the evaluated class, their decision is accepted; otherwise, a postclassifier is employed to make the final decision. A method for combining multiple sources based on their classification accuracies has been proposed by Lisini et al. [21]. In this context, some methods proposed utilizing fuzzy aggregation rules as well as fuzzy set theory and fuzzy fusion to deal with the uncertainty of the classifier's output [22, 23].

For the purpose of age estimation based on speech data, we employ fuzzy data fusion in the current study in order to aggregate the decisions made by a few classifiers. A “divide and conquer” strategy is employed, in which the processing speech data are divided into some groups based on the vowel classes. There are two reasons behind this strategy. First, decreasing the complicated distribution of the processing data improves the classifier's learning performance. Second, different vowel classes contain complementary sets of information for age estimation. In the next step, the classifiers are applied on each group to make a primary decision. Subsequently, fuzzy data fusion is employed to provide an overall decision by aggregating the classifier's outputs. The rest of the paper is organized as follows.  Section 2 presents the feature extraction for the proposed method, Section 3 discusses the self-adaptive extreme learning machine (SaELM) learning and support vector machine (SVM) for classification, Section 4 presents the fuzzy fusion and relevant theory, Section 5 presents the experiments, and Section 6 concludes the paper.

## 2. Feature Extraction

In pattern recognition, the extraction of meaningful low-dimensional representation from the given data with higher dimensions is a procedure known as feature extraction. One of the most frequently used feature extraction methods in ASR approaches is MFCC [24]. In this method, a Mel filter bank is employed to represent the human auditory model. In computing MFCCs for most ASR approaches, 13 triangular Mel filters are used to produce the cepstral coefficients based on discrete cosine transform. Afterwards, 13 delta and 13 delta-delta coefficients are added to the static cepstral features to represent the temporal information of speech samples. The spectral smoothing performed by the Mel filters may eliminate some relevant information for age estimation; thus, narrower Mel filters are used in the current study. Consequently, a higher number of static Cepstral features (40 in this study) are obtained. Then delta and delta-delta coefficients are added to the feature vector. Using this strategy, lower spectral smoothing is applied using the Mel filters.

## 3. Classification

In this section, SVM and SaELM classification methods that are employed in this study are explained.

### 3.1. Support Vector Machine (SVM) for Classification

The support vector machine (SVM) introduced by Vapnik in 1998 is a binary classification method based on the notion of maximum margin between classes. It performs based on structural risk minimization (SRM) theory [25] and has been revealed as a powerful tool for various pattern classification problems [26]. To introduce SVM let *X* = {*x*_1_, *x*_2_,…, *x*_*n*_} denote training data set of two classes. An indicator vector *y* is definable as

(1) $$\begin{array}{c} y_{i}\{\begin{array}{cc} 1 & \text{if}\;\;x_{i}\;\;\text{in}\;\;C_{1}\\ -1 & \text{if}\;\;x_{i}\;\;\text{in}\;\;C_{2}, \end{array} \end{array}$$

and decision function is

(2) $$\begin{array}{c} d\left(x\right)=\mathrm{sign}\left(w^{T}x+b\right), \end{array}$$

where *w* and *b* denote the weight vector and the bias, respectively. The main idea of SVM includes maximization of the margin between the closest vector and the hyperplane. Consequently, the optimal separating hyperplane is obtainable by solving the following quadratic problem:

(3) min⁡ 12wTwsubject  to yi(wTx+b)≥1.

In some real world classification problems data are not linearly separable. As a remedy for this problem, kernel-based transformation is employed to map the input data space to a higher dimensional space that the training data is separable. The most frequent kernel functions are the Gaussian radial basis function (RBF), polynomial kernel, and linear kernel. In this paper, linear kernel is used for the kernel function.

### 3.2. Self-Adaptive Extreme Learning Machine for Classification

Along with the frequent usage of SVM in many pattern recognition approaches [27], neural networks are also potential alternatives to SVM in some multiclass classification applications. Although conventional neural networks have some deficiencies, such as higher computational time along with classification accuracy problems, an efficient cure has been proposed for this problem by Huang et al. [28]. Their method comprises a learning algorithm called extreme learning machine (ELM) for single hidden layer feedforward neural-networks (SLFNs). In this method, input weights of the SLFN are randomly selected and the output weights are analytically computed. To explain the ELM algorithm, we first define the standard SLFN. Suppose that we have *n* samples (*x*_*i*_, *t*_*i*_) representing *p*-dimensional feature vectors *x*_*i*_ = [*x*_*i*1_, *x*_*i*2_,…, *x*_*in*_]^*T*^ ∈ *R* ^*n*^ and the target vector *t*_*i*_ = [*t*_*i*1_, *t*_*i*2_,…, *t*_*im*_]^*T*^ ∈ *R* ^*m*^, respectively. Consequently, a standard SLFN with *N* hidden neurons and activation function *g*(*x*) can be expressed as follows:

(4) $$\begin{array}{c} \overset{\tilde{N}}{\underset{i=1}{\sum }}\beta_{i}g\left(w_{i}\cdot x_{j}+b_{i}\right)=o_{j},\;j=1,\ldots ,N, \end{array}$$

where *w*_*i*_ = [*w*_*i*1_, *w*_*i*2_,…, *w*_*in*_]^*T*^ denotes the weight vector that connects *i*th hidden neuron and input neurons; *β*_*i*_ = [*β*_*i*1_, *β*_*i*2_,…, *β*_*im*_]^*T*^ is the weight vector that connects the *i*th neuron and output neurons; and *b*_*i*_ is the threshold of the *i*th neuron. The “·” in *w*_*i*_ · *x*_*i*_ denotes the inner product of *w*_*i*_ and *x*_*j*_. SLFN aims to minimize the difference between *O*_*j*_ and *t*_*j*_. This can be expressed mathematically as follows:

(5) $$\begin{array}{c} \overset{\tilde{N}}{\underset{i=1}{\sum }}\beta_{i}g\left(w_{i}\cdot x_{j}+b_{i}\right)=t_{j},\;j=1,\ldots ,N. \end{array}$$

In other words we have *Hβ* = *T*, where

(6) H(w1,…,wN~,b1,…,bN~,x1,…,xN)  =[g(w1·x1+b1)⋯g(wN~·xN~+bN~)⋮⋯⋮g(w1·xN+b1)⋯g(wN~·xN+bN~)]N×N~,β=[β1T⋮βN~T]N~×m,  T=[T1T⋮TN~T]N×m.

As proposed by Huang et al. [28], *H* here is called the neural network output matrix. ELM algorithm operates as follows [29].

Given a training set

(7) $$\begin{array}{c} N=\left\{\left(x_{i},t_{i}\right)\;|\;x_{i}\in R^{n},\;\;t_{i}\in R^{m},\;\;i=1,\ldots ,N\right\}, \end{array}$$

(1) allocate random value to the input weight *w*_*i*_ as well as the bias *b*_*i*_, $i=1,\ldots ,\tilde{N}$;
(2) compute the hidden layer output matrix *H*;
(3) compute the output weight *β* as follows:
  (8) $$\begin{array}{c} \beta =H^{+}T, \end{array}$$

where *β*, *H*, and *T* have similar definitions as the SLFN parameters expressed above.

As discussed before, SLFN aims to minimize the difference between *O*_*j*_ and *t*_*j*_ and the ELM algorithm allocates random values to the input weights and the bias, subsequently from (8), is computed. After proposing the basic ELM, some researchers suggested some strategies to generate the random values for *β* and *H* to obtain a global minimum for the minimization problem mentioned above. Evolutionary ELM [30] and self-adaptive ELM [31] are the proposed algorithms that employed evolutionary methods for finding the optimal parameters for ELM. E-ELM performed better than basic ELM but choosing an appropriate trial vector generation strategy was a potential problem for this method. Therefore, self-adaptive ELM was proposed later which incorporated the self-adaptive differential evolution algorithm [32] to optimize the network input weights and hidden node biases and the extreme learning machine to derive the network output weights. Comparative experiments with SVM in previous works have revealed that this method outperformed SVM in many classification problems and obtained better generalization performances than several related methods [31]. Thus, we use this method in the current study for the purpose of classification.

## 4. Fuzzy Information Fusion

### 4.1. Fuzzy Set Theory

Based on traditional mathematics, the possible membership of an element to a set can be defined as a crisp value of 0 or 1, such that the membership is 1 for an element that is a member of the set and 0 otherwise. In contrast to the traditional mathematics, “fuzzy set” theory, first introduced by Zadeh [33], provides the idea of partial membership to a set. The membership is a real value in a range of zero and 1. This theory has been proposed to resolve modeling of vagueness as well as ambiguity in various systems. One of its valuable advantages is its capability to deal with uncertain data in complex problems, such as postprocessing of outputs provided by a group of classifiers. To explain this theory, we use the notations in a previous work [34].

Let *A* be a mapping from *X* (an ordinary nonvoid set) into the interval [0, 1]. The value *A*(*x*) of *A* in *x* ∈ *X* indicates the degree of membership of *x* in *A*. The set of all elements that have a nonzero degree of membership in *A* is called the support of *A*, which is given by

(9) $$\begin{array}{c} \text{SUPP}\left(A\right)=\left\{x\;|\;x\in X,\;\;A\left(x\right)>0\right\}. \end{array}$$

The set of elements that completely belong to *A* is called the kernel of *A* and is given by

(10) $$\begin{array}{c} \ker\left(A\right)=\left\{x\;|\;x\in X,\;\;A\left(x\right)=1\right\}. \end{array}$$

The set of elements having the largest degree of membership in *A* is called the core of *A*, which is expressed as

(11) $$\begin{array}{c} \text{core}\left(A\right)=\left\{x\;|\;x\in X,\;\;\neg \left(\exists y\in X\right)\left(A\left(y\right)>A\left(x\right)\right)\right\}. \end{array}$$

The weak  *α*-*cut*, in a fuzzy set *A* on *X* is defined as the set of all elements of *X* whose degree of membership in *A* is at least equal to *α*, where *α* ∈ [0, 1]. The weak *α*-*cut* in a fuzzy set *A*, *A*_*α*_, is given as follow

(12) $$\begin{array}{c} A_{\alpha }=\left\{x\;|\;x\in X,\;\;A\left(x\right)\geq \alpha \right\}. \end{array}$$

Defuzzification is expressed by a defuzzification operator *D*. This operator maps fuzzy sets on *X* into elements of the universe *X* expressed as

(13) $$\begin{array}{c} D:F\left(X\right)⟶X:A⟶D\left(A\right). \end{array}$$

### 4.2. Problem Definition

Let us suppose an *n*-class classification problem provided by *m* different classifiers. For a given speech sample *x*, the output of classifier *i* is the set of numerical values given by

(14) $$\begin{array}{c} \left\{\mu_{i}^{1}\left(x\right),\mu_{i}^{2}\left(x\right),\ldots ,\mu_{i}^{n}\left(x\right)\right\}, \end{array}$$

where *μ*_*i*_^*j*^(*x*)∈[0, 1] denotes membership degree of sample *x* to class *j* provided by classifier *i*. The higher this value is, the more likely it is that the speech sample fits class *j*. Based on the classifier, *μ*_*i*_^*j*^(*x*) can be represented by probability, posterior probability at the output of a neural network, membership degree at the output of a fuzzy classifier, and so on. Consequently, the set *π*_*i*_(*x*) = {*μ*_*i*_^*j*^(*x*), *j* = 1,…, *n*} can be considered as a fuzzy set. In speech processing context, for each speech sample (feature), *m* fuzzy sets are provided. Therefore, the inputs for fusion procedure include {*π*_1_(*x*), *π*_2_(*x*),…, *π*_*i*_(*x*),…, *π*_*m*_(*x*)}.

### 4.3. Information Fusion Based on Fuzzy Aggregation

Combining different sources of information to improve the overall decision, also known as information fusion, is an effective way to cope with decision making under conflicting circumstances. After formulating the uncertain data, including decision of classifiers into the fuzzy sets, fuzzy aggregation is required to achieve an overall decision. In order to aggregate the fuzzy sets, numerous combination operators have been proposed in literature, in which each operator has its own properties that can be useful depending on the in-hand problem. The operators are categorized in three groups as follows:

1. conjunctive combination,
2. disjunctive combination,
3. compromise combination.

#### 4.3.1. Conjunctive Combination

This kind of aggregation results in a set that is unavoidably smaller than the initial set. T-norms are of this kind. The following properties are satisfied with conjunctive combinations given by

(15) $$\begin{array}{c} \pi_{\text{CC}}\left(x\right)\leq \underset{i\in \left[1,m\right]}{\min}\pi_{i}\left(x\right), \end{array}$$

where *π*_CC_(*x*) denotes the results of combining the sets, which leads to

(16) $$\begin{array}{c} \left(\pi_{\text{CC}}\left(x\right)=\pi_{1}\left(x\right)\circ \pi_{2}\left(x\right)\circ \cdots \circ \pi_{m}\left(x\right)\right). \end{array}$$

#### 4.3.2. Disjunctive Combination

This kind of aggregation results in a set that is inevitably larger than the aggregating sets. T-conorms are instants of this kind of aggregation operator. The following properties are satisfied with this kind, which is given by

(17) $$\begin{array}{c} \pi_{\text{CC}}\left(x\right)\geq \underset{i\in [1,m]}{\max}\pi_{i}\left(x\right). \end{array}$$

#### 4.3.3. Compromise Combination

Compromising of the aggregating set is performed based on this kind of aggregation operator. For instance, in *π*_CC_(*x*), the compromise combination of *π*_1_(*x*) and *π*_2_(*x*) satisfies the following property:

(18) $$\begin{array}{c} \min\left(\pi_{1}\left(x\right),\pi_{2}\left(x\right)\right)<\pi_{\text{CC}}\left(x\right)<\max\left(\pi_{1}\left(x\right),\pi_{2}\left(x\right)\right). \end{array}$$

Based on a classification proposed by Bloch in 1996, these operators are recognized as contextual dependent (CD) operators [35]. There are different criteria to distinguish the context in our problem, including the information about possible conflicts between the sources and the reliability of each source. The operators have been introduced under the possibility theory [36], but they are applicable in fuzzy set theory as well. Here, considering the context, the operators are adapted to deal with the fusion of the classifier's output. Fauvel et al. [22] proposed some suggestions for using the combination operators based on the conflictions among sources. They recommended using the conjunctive, disjunctive, and compromise combination operators for dealing with low, high, and partial conflictions of the sources, respectively. In addition to the information regarding the confliction of the sources, their reliability should be formulated into the CD operator to enable them to handle the problem effectively. In Section 4.4.3 we show how we use reliability of the classifiers, which is known here as context, to perform classifier fusion.

### 4.4. Obtaining the Classifier's Decisions and Confidence Measurement

As previously mentioned, combining different sources of information to improve the overall decision is the idea behind the current study. Different vowels uttered by each speaker provide diverse sources of information, which are employed for estimation of speaker's age. Dealing with the age estimation problem, two different classification scenarios are studied including vowel-based age estimation and vowel independent age estimation methods. The former method is employed for classifier fusion while the latter method is only used for comparison to the fusion method.

#### 4.4.1. Vowel-Based Age Estimation Accuracy

In this part, before applying the age estimation, the database was separated based on the vowels. In other words, training and testing were performed separately for each vowel. Therefore, the number of the age estimation accuracies provided in this section was set to be equal to the number of the vowel classes. Outputs of the classifiers were collected to measure the confidence of each decision made by the classifier.

(*1) Local Confidence Measurement versus Global Confidence Measurement for Each Classifier*. For each testing sample, output of each classifier includes six log-probabilities, which present membership of the sample to the age classes. Based on the log-probabilities a sample-based confidence is computed known as local confidence coefficient. Additionally, after processing all of the samples by a classifier, ability of the classifier in recognition of the samples of each class is computable. This ability is referred to as global confidence. For example, suppose that a classifier recognizes the samples from “Class 7” with the highest accuracy in comparison with other classifiers. Consequently, the global confidence of the classifier in recognizing the samples in “Class 7” is higher than that of others. In this study for a specific class, the global confidence of a classifier with the highest confidence is set to one and global confidence of other classifiers is set to zero. For obtaining the global confidence for each classifier only training data are employed. Based on leave-one-out cross validation method performed on the training samples, the global confidence is computed for each classifier.

#### 4.4.2. Vowel Independent Age Estimation Accuracy

Here, the classifiers were trained with the entire training database, including all of the vowels. In other words, each speech sample for age estimation is one of the vowels uttered by a speaker. Consequently, the number of employed samples in this section is 6 times that of the previous section, but the number of the features in each speech sample is one-sixth that in previous section. Based on this classification scenario, vowel independent age estimation accuracy was obtained.

#### 4.4.3. Combination Operator and Decision Fusion

A large number of combination operators have been proposed in literature. The combination operator we used in this study is known as “fuzzy-or” operator. It is a compromise combination operator expressed as

(19) μfj(x)=γmax⁡i=1m(min⁡(wiμij(x),δij)) +(1−γ)1m∑i=1mwiμij(x)δij,

where *μ*_*i*_^*j*^(*x*) denotes the* j*th output of the *i*th classifier, which is normalized according to outputs of *i*th classifier; *w*_*i*_ is the local confidence coefficient associated with the classifier's output; *δ*_*i*_^*j*^ is the global confidence coefficient; *μ*_*f*_^*j*^ denotes the fusion result; and *γ* is the compensation degree. For *γ* = 1, the fuzzy-or operator behaves as max-operator, and the behavior of the operator for *γ* = 0 is similar to the arithmetic average of the fuzzy memberships. The confidence coefficient, *w*_*i*_, represents the reliability of each classifier's output for a given test sample. Here, *w*_*i*_ can be obtained as follows:

(20) wi(x)=exp⁡(−0.5(|1−((Smax⁡1−Smax⁡2)/(Smax⁡1−Smin⁡))|σ)2),

where *S*_max⁡1_, *S*_max⁡2_, and *S*_min⁡_ are the highest, second highest, and lowest amounts in the output vector, respectively, which are produced by *i*th classifier, [*μ*_*i*_^1^, *μ*_*i*_^2^,…, *μ*_*i*_^*n*^]. In addition, *σ* is the standard deviation of the Gaussian membership function. As (20) indicates, for a given test sample, the decision of a classifier is reliable if the highest output representing the classifier's decision is considerably higher than other outputs of the classifier. Consequently, *w*_*i*_ takes a higher value for reliable classifiers.

**Figure 1:**
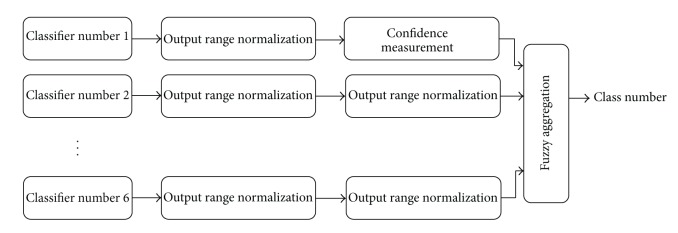
Block diagram of the proposed fuzzy data fusion method.

After performing fusion of the decisions provided by the classifiers based on (19), a vector representing the overall decision is obtained. The highest value in the vector presents the winner class assigned to the test sample. Note that the fusion strategy aggregates complementary information from different sources of speech for the age classification problem.  Figure 1 presents the block diagram of the proposed fusion method.

#### 4.4.4. SVM Based Vowel Classification

In order to perform age classification in a fully automated manner, a SVM based vowel classifier with a linear kernel is developed for age classification to divide the testing samples into the vowel classes. Before dividing the test samples vowel classifier is trained with the training samples of the age classifier. Note that the only difference between the age classifiers and the vowel classifier is the training labels that show the vowel class to the vowel classifier. Based on this technique, without having prior phonetic knowledge of a testing sample its age class can be predicted.

## 5. Experimental Results

In this section we present experiments conducted to benchmark the proposed age estimation method. For this purpose a speech database from children has been collected for age estimation. After applying the proposed method to the speech corpus, for evaluating the merit of the proposed method, a comparison to the other age estimation methods was carried out.

### 5.1. Speech Corpus

Three hundred sixty normal Malaysian children aged between 7 and 12 participated in this study. Each age group (grouped by calendar) consisted of 30 males and 30 females. All subjects were selected from primary schools in Malaysia. None of them had vocal pathology or voice disorder, symptoms of cold or flu, allergies, history of smoking, neurologic disease, or respiratory dysfunction. The subjects were asked to pronounce sustained Malay vowels of /a/, /e/, /*ə*/, /i/, /o/, and /u/ for 5 s each at a comfortable pitch and loudness level. The speech sounds were recorded using a Shure SM58 microphone in a regular room environment. The mouth-to-microphone distance was fixed at 2-3 cm. Gold-Wave digital audio editor software was used to record the speech sounds at a sampling rate of 20 kHz with 16-bit resolution.

The speech database is summarized in Table 1.

A discrimination test was administered to check the pronunciation of the vowels before extracting the fundamental and formant frequency values. Ten students from University of Malaya listened to the samples and participated in the discrimination test. They listened to all the recorded sustained vowels of the children and identified the vowel they heard. The pronunciation of the vowels was considered correct if seven of the 10 listeners identified them correctly.

### 5.2. Experimental Setup

The single-frame feature extraction method was used to extract MFCC from the speech samples. The frame length for this method was 55 ms. For each speech sample, 120 MFCCs were computed, including 40 static, 40 delta, and 40 delta-delta coefficients. Experiments were accomplished based on a 3-fold cross validation method. In this method two-thirds of the same database was used to train the SaELM and SVM, while the remaining one-third of the database was used for the validation. This experiment was repeated three times based on three different training and test sets. The training set and the test set were not in common. The recognition rates obtained from the three test sets were averaged. Neural networks based on the SaELM method and different activation functions as well as different numbers of hidden neurons were used for classification. Moreover, a number of experiments were used to adjust the SaELM parameters for the experiments. The mutation strategy employed in SaELM was “DE/rand-to-best/2” strategy (see [31] for more details). The positive amplification factor was set to 1 and the crossover rate parameter was set to 0.5. 40 populations in each generation of the evolutionary ELM were used and 15 generations were employed for evolution. Based on the experiments, best number of hidden neurons for the ANN was 60.

The experiments were conducted in three parts. In the first part, the classifier was applied to the samples from all of the vowels. In the second part, the speech samples were divided into six groups based on the uttered vowels before performing the classification. The classifiers were applied to the groups to evaluate the age of the speakers based on different vowels. Note that, for testing the samples, prior to the age classification, the samples were phonetically classified by a SVM based vowel recognizer. Meanwhile, the outputs of the classifiers were collected for the third part. In the last part, the fusion of the decisions provided by the classifiers in the previous parts was performed.

### 5.3. Age Estimation of the Speakers Uttered Different Vowels

In this part, ANN method based on SaELM training was applied to the speech database, which contained samples from the entire set of phonemes. As a comparison to other well-known classification methods in literature, similar experiments were performed using the SVM and KNN methods. For this purpose, SVM method with different kernels and KNN method with different neighborhoods were applied to the database, after which the best accuracies provided with the methods were recorded.  Table 2 summarizes the results.

### 5.4. Vowel-Based Age Estimation

In this part of the experiment, which was performed before the classification, the database was divided into the vowel groups. Then SaELM method was applied to each group in order to perform the age estimation. Meanwhile, different activation functions were used for the classifier. In a neural network, activation functions include combination function and transfer functions that pass the input and hidden nodes to the hidden and output layers, respectively, through a nonlinear/linear function. In this part of the experiment, different activation functions, including sin, sigmoid, and Hardlim functions, were used for the ANN. The best accuracy was obtained by using the Hardlim activation function.  Table 3 presents the summary of the vowel-based age estimation results.

### 5.5. Fusion of the Classifier's Decisions

After collecting the decisions of the classifiers from the previous part, an overall decision can be made by fusing the classifier's outputs. The fusion of the decisions was performed using the fuzzy method discussed in Section 4.4.3. Here, *σ* in the confidence coefficient was 0.05 and the compensation degree was 0.6.  Table 3 presents the fusion results. As can be seen, considerable improvement of age estimation is achieved by applying the fusion (Table 3). The results show that different vowels reflect complementary information regarding age estimation.

Dividing the speech data into vowel groups can decrease the complexity of data distribution in *n*-dimensional feature space. Therefore, classifiers can be more effectively trained on each group of the vowels. Meanwhile, the fuzzy formulation of the uncertainties of the classifier's output could help realize this objective. The novelty of our approach lies in aggregation of complementary information from both different sources of data and different classification methods based on the fuzzy fusion method. Moreover, SVM based vowel classifier across with the proposed age estimation method provided ability of predicting the sample's age without having priori phonetic knowledge of the sample. In other words the phonetic and the age of the samples are recognized with the system.

Table 4 presents the confusion matrix of the proposed age estimation method. As can be seen, the highest and lowest accuracies are obtained for ages 7 and 11, respectively (Table 4). In some applications, age estimation is also acceptable in wider age groups including the 7-8, 9-10, and 11-12 age groups. Based on this definition, a new confusion matrix has been computed (Table 5). As can be seen, the overall age estimation accuracy is 60.83%, and the age group including the 7-8 groups provides the accuracy of 90.0%.

### 5.6. Comparisons with Other Age Estimation Methods

For the purpose of comparison, two state-of-the-art age estimation methods proposed by Mahmoodi et al. [12] and Bahari et al. [17] were simulated and applied to the speech database for age estimation.

Similar to the proposed method, the speech samples from different vowels uttered by each subject have been used to make a large feature vector. Consequently, same amount of information as the proposed method has been fed to the baseline systems for age estimation.  Table 6 presents the comparison of the results. As Table 6 shows the proposed method outperformed the baseline methods because despite employing equal amount of acoustic information from each subject, the proposed method decreased the complexity of the processing data in *n*-dimensional feature space which improved learning of the classifiers employed for age estimation problem.

## 6. Conclusion

The fusion of several classifiers trained by different sources has been considered for estimating speaker's age in the current work. In order to reduce the complexity of the data distribution in *n*-dimensional feature space, the speech data has been divided into six vowel groups. Afterwards, vowel-based age classification has been performed to process the data. SLFNs trained by SaELM are also used for classification. Speech data included 6 Malay vowels uttered by 360 children aged between 7 and 12 years. Subsequently, fuzzy information fusion is used to provide decision fusion of the classifiers trained in the previous step. The overall accuracy of the decision fusion reveals a considerable improvement compared with the classification accuracy of each group or vowel independent classification. The fuzzy aggregation of complementary information, which is collected from different classifiers, provides a rich source of data for age estimation analysis.

## Figures and Tables

**Table 1:** Summary of speech database.

Speaker ages	/a/	/e/	/*ə*/	/i/	/o/	/u/
7	60	60	60	60	60	60
8	60	60	60	60	60	60
9	60	60	60	60	60	60
10	60	60	60	60	60	60
11	60	60	60	60	60	60
12	60	60	60	60	60	60

**Table 2:** A comparative result of vowel independent age estimation.

Classification method	Accuracy (%)	Specifications
ANN (ELM)	24.77	100 hidden neurons
SVM	24.21	Linear kernel
KNN	23.47	Euclidean distance, number of nearest neighbors = 20

**Table 3:** Vowel-based age estimation accuracy (in percentage) based on different activation functions and fusion of the results using the proposed fuzzy information fusion method.

Vowel groups						Fusion
/a/	/e/	/*ə*/	/i/	/o/	/u/	
25.83	23.33	29.17	25.83	19.17	30.83	53.33

**Table 4:** Confusion matrix of the proposed age estimation method based on 6 age classes.

	7	8	9	10	11	12	Accuracy (%)
7	17	1	2	0	0	0	85.0
8	3	15	1	1	0	0	75.0
9	5	1	11	2	0	1	55.0
10	6	5	1	6	1	1	30.0
11	4	4	1	1	9	1	45.0
12	7	3	1	2	1	6	30.0
							53.33

**Table 5:** Confusion matrix of the proposed age estimation method based on 3 age classes.

	7, 8	9, 10	11, 12	Accuracy (%)
7, 8	108	12	0	90.0
9, 10	51	60	9	50.0
11, 12	54	15	51	42.5
				60.83

**Table 6:** A comparative result of the proposed method and the baseline system for age estimation.

Classification method	Accuracy (%)	Specifications
Proposed method	53.33	60 hidden neurons
SVM [12]	30.56	Linear kernel, Gamma = 2
*i*-vector and SVR [17]	37.5	Supervector size = 300, linear kernel, Gamma = 2
